# Effect of autologous platelet-rich plasma on new bone formation and viability of a Marburg bone graft

**DOI:** 10.1515/biol-2022-0761

**Published:** 2023-11-21

**Authors:** Dina Saginova, Elyarbek Tashmetov, Berik Tuleubaev, Yevgeniy Kamyshanskiy

**Affiliations:** The Center for Applied Scientific Research, National Scientific Center of Traumatology and Orthopaedics Named After Academician N.D. Batpenov, Astana 010000, Kazakhstan; Department of Surgical Diseases, Karaganda Medical University, Karaganda 100000, Kazakhstan; Pathology Unit of the University Clinic, Karaganda Medical University, Karaganda 100000, Kazakhstan

**Keywords:** bone graft, bone defect, PRP, Marburg bone bank system

## Abstract

This study aimed to compare the new bone formation, the process of remodeling, and the viability of bone grafts, using a combination of platelet-rich plasma (PRP) and Marburg bone graft versus bone grafts without any additional elements. For this study, 48 rabbits (with 24 rabbits in each group) were used. Bone defects were made in the femur, and the bone graft used was the human femoral head prepared according to the Marburg Bone Bank. Rabbits were divided into the following groups: heat-treated bone graft (HTBG group) and HTBG with PRP (HTBG + PRP group). After 14, 30, and 60 days post-surgery, the assessment of the results involved X-ray, histopathological, and histomorphometric analyses. The greater new bone formation was detected in the HTBG + PRP group on the 14 and 30 day (*p* < 0.001). Furthermore, the group using bone grafts with PRP demonstrated notably enhanced remodeling, characterized by stronger bone integration, more significant graft remineralization, and a circular pattern of newly formed bone. The PRP–bone graft complex improves bone tissue repair in the bone defect in the initial stages of bone regeneration. PRP has been identified to enhance the remodeling process and amplify the osteoconductive and osteoinductive capabilities of HTBGs.

## 1 Introduction

Bone defects commonly manifest as a result of bone resection, metabolic pathologies, traumatic events, and neoplasms. Annually, these osseous deficiencies necessitate more than one million reconstructive surgical interventions [1,2]. The main principle of treatment of defects of any etiology is their filling with bone substitutes. Autologous bone is the “gold standard” [3,4]. First, this is due to the fact that the autologous bone does not cause any immunological reactions. Second, it has simultaneously both osteoinductive and osteoconductive properties – the former is represented by bone cells and growth factors. The limited availability of autologous bone and the accompanying trauma induced by the harvesting procedure restrict its use in clinical practice [3,4,5]. Synthetic bone grafts serve as an alternative to autologous graft options due to their extensive accessibility, gradual biodegradation, and lack of risks such as donor-site morbidity and viral transmission [6–9]. Yet, their biological role is constrained, particularly in fracture healing, because of their purely osteoconductive nature, mechanical properties, and absence of angiogenic attributes [1,6,10,11]. On the other hand, allogenous bone grafts, sourced from humans or human cadavers, are subject to sterile processing before transplantation to a recipient. The osteoconductive and occasionally osteoinductive potential of these grafts can vary based on the preparation process [1,3,7].

The Marburg Bone Bank-prepared bone graft is a type of bone allograft widely used in orthopedic surgery. These grafts are extensively processed to remove cellular material, leaving behind only the mineralized bone matrix. The processed bone matrix provides a scaffold for new bone growth, which can lead to faster and more complete healing [12–15]. Nevertheless, the osteoinductive capability of processed (chemically or physically) allogeneic bone is not clearly established, given that osteogenic cells are eradicated during the tissue manipulation process. This leads to the partial retention of osteoinductive substances, which may contribute to suboptimal clinical effects. Currently, there has been a surge in interest regarding the use of growth factors and morphogens as potential agents for conferring osteoinductivity to bone substitutes [16–18].

Platelet-rich plasma (PRP) obtained from autologous blood represents a significant resource for delivering high levels of growth factors, including platelet-derived growth factor (PDGF), transforming growth factor beta, epidermal growth factor, vascular endothelial growth factor, and insulin-like growth factor I. These factors are vital in tissue growth and progression, and their introduction to the site of bone defects is crucial [19,20]. PRP has shown potential for use in combination with bone grafts, as *in vitro* studies have demonstrated a notable increase in osteoblast proliferation upon the addition of PRP [19]. Furthermore, incorporating PRP with graft materials has been shown to reduce the duration required for graft consolidation, maturation, and improvement of trabecular bone density [20–23]. These findings suggest that PRP may compensate for the limited osteogenic potential of thermally disinfected bone grafts to promote osteogenesis in bone defects. However, there is limited information regarding the synergistic effects of PRP and Marburg-prepared bone graft on bone healing. Therefore, the purpose of this study is to perform a comparative assessment of the new bone formation, the process of remodeling, and the viability of bone grafts, using a combination of PRP with bone grafts prepared by the Marburg Bone Bank System versus bone allografts without any additional elements.

## 2 Methods

### 2.1 Preparation of Marburg bone grafts

**Figure 1:**
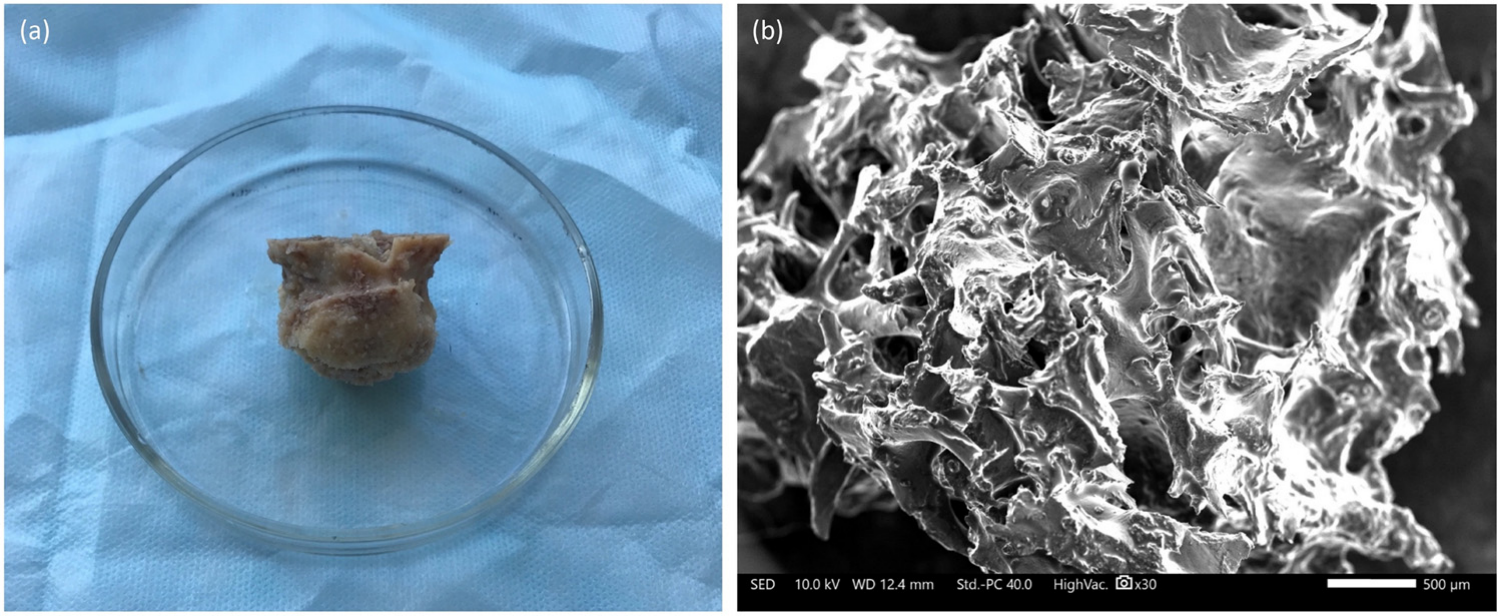
Heat-treated femoral head (a) and scanning electron microscopic view of bone graft (b).


In this study, femoral heads prepared by the Marburg Bank were used as bone graft (Figure 1). These femoral heads were obtained from a living donor who had undergone hip joint arthroplasty surgery in compliance with national regulations [24,25]. During hip joint endoprosthetics, the head of the femur is removed from the operating room and subjected to a series of mechanical cleaning procedures in sterile conditions to eliminate any soft tissue, cartilage, and ligaments. The femoral heads were placed in a disposable sterile container and filled with 0.9% NaCl solution in a volume of 300 ml, and then they were sealed and subjected to a heat treatment process in a Lobator SD-2 (Telos Company, Germany) device for a total of 94 min, maintaining a temperature of 82.5°C in the femoral head for at least 15 min, according to the established protocol. The sterility of the container was verified at the end of the cycle through a specific opening, and then, the liquid was completely drained. The bone allografts were then stored in a freezer at −80°C as per the recommended protocol [13,26]. Two hours before the experiment, the femoral head was unfrozen at room temperature and cut into chips. To ensure a consistent ratio of ingredients, a specific weight was used to standardize the mixture of bone graft with PRP 0.5 g bone allograft/0.5 ml PRP.

**Informed consent:** Informed consent has been obtained from all individuals included in this study.
**Ethical approval:** The research related to human use has been complied with all the relevant national regulations, institutional policies and in accordance with the tenets of the Helsinki Declaration, and has been approved by the authors' institutional review board or equivalent committee.

### 2.2 Preparation of PRP

Prior to each surgical intervention (approximately 30 min before transplantation), 5 ml of blood was collected from the heart into siliconized tubes containing 3.8% sodium citrate at a blood/citrate ratio of 9:1 [27]. PRP was procured via two-step centrifugation [28]. Initial centrifugation involved the separation of blood cell elements using a laboratory centrifuge, with tubes centrifuged at 900 G for 8 min at ambient temperature, yielding two primary components: the blood cell component (BCC) in the lower fraction and in the upper fraction. Subsequent centrifugation entailed marking a point 6 mm beneath the line demarcating the BCC. To augment the total platelet count for the second centrifugation, all contents above the mark were transferred to another 5 ml vacuum tube devoid of anticoagulant. The sample was then centrifuged once more at 1,500 G for 5 min to obtain two components: platelet-poor plasma (PPP) and PRP. Approximately 0.5 ml of PRP was separated from the PPP. The PRP (0.5 ml) was combined with 0.5 g of bone graft and used to fill the created defect.

### 2.3 Animals and surgical procedures

In this investigation, 48 outbred rabbits were acquired, comprising adult males (6–7 months old) with an average weight of 3,025 ± 87 g. The animals were housed in cages and allowed to acclimate for a duration of 2 weeks. All procedures involving the animals adhered to the protection guidelines of DIRECTIVE 2010/63/EU for animals used in scientific research [29] and were approved by the University Animal Care Committee (UACC) under protocol № 27 dated 27.09.2020. Throughout the study, the rabbits were maintained at room temperature (22 ± 2°C), with 40–50% humidity, and subjected to a 12 h light–dark cycle. The animals were provided with standard rabbit pellets and tap water for sustenance.

Following Russell and Burch’s bioethical principles of replacement, reduction, and refinement, the sample size for animal experimentation in this study was established as the minimum number of animals necessary to yield statistically significant outcomes [30].

**Figure 2:**
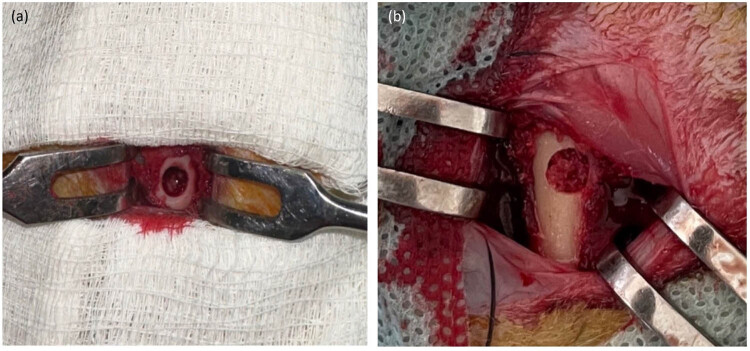
Bone defect in rabbit femur (a) and bone defect after filling with HTBG (b).


The rabbits were randomly allocated into two groups (*n* = 24 per group) and underwent the same surgical procedure. Three hours before, the rabbits received an intramuscular (i.m.) injection of gentamycin 0.1 ml/kg (Mapichem, Switzerland). Animals were anesthetized with an i.m. injection of Zoletil 0,1 mg/kg (Virbac, USA) and Rometar 5 mg/kg (Bioveta, Czech Republic). Following hip skin disinfection, an incision was made in the distal femur. The periosteum was then lifted, and a 5 mm diameter unicortical bone defect was created in the distal femoral metaphysis using a drill (Figure 2) [31]. Then, based on the experimental group assignment, the bone defects in the first group were treated with a heat-treated bone graft (HTBG group), whereas in the second group, the defects were treated with the HTBG with autologous PRP (HTBG + PRP group). The surgical wound was sutured with absorbable sutures (5-0 Vicryl, Ethicon, Johnson & Johnson, USA). After surgery, each animal received i.m. injections of gentamycin 0.1 ml/kg (Mapichem, Switzerland) and ketonal 0.04 ml/kg (Sandoz, Slovenia) for 3 days. Daily postoperative observations were made to monitor the healing progress according to a predetermined schedule for consecutive days. There were no complications or deaths in the postoperative period. At 14, 30, and 60 days, 8 animals from each group (16 rabbits for each time period) were sacrificed according to ethical standards by intravenous administration of lethal doses of Zoletil 50 mg/ml and the distal femur was harvested.

**Ethical approval:** The research related to animal use has been complied with all the relevant national regulations and institutional policies for the care and use of animals and has been approved by the UACC.

### 2.4 Histopathological examination

Prior to histological analysis, the specimens were fixed in 10% neutral buffered formalin for a duration of 24 h, followed by decalcification in Biodec R solution (Bio-Optica Milano SPA) for an additional 24 h. Subsequently, the samples were rinsed in phosphate buffer (pH = 7.4). Upon achieving optimal bone tissue softening (decalcification), a bone incision was executed. The tissue was then fixed in 10% formalin at 4°C for 24 h, washed with tap water, and dehydrated using a series of ascending alcohol concentrations (70, 90, 95, and 100%). The samples were then immersed in xylene and embedded in paraffin blocks. Tissue sections with a thickness of 5 μm were prepared using a Leica SM 2000R sliding microtome. Once prepared, the tissue sections were treated with hematoxylin and eosin for overall tissue morphological analysis, identification of inflammatory infiltrate and necrosis, and Masson’s trichrome to evaluate bone graft remodeling and new bone formation [32].

#### 2.4.1 Hematoxylin and eosin staining process

The tissue slices were submerged in Mayer’s hematoxylin for a quarter of an hour, followed by a 5 min rinse with water. Thereafter, the sections were subjected to a minute-long eosin staining.

#### 2.4.2 Masson’s trichrome staining procedure

A commercial kit (Trichrome Stain (Masson) Biovitrim TU 9398-001-89079081-2012) was used for Masson trichrome staining. After dewaxing and rehydration, the slide specimens were placed in Bouin’s solution at 56°C for a duration of 15 min. This was succeeded by a 5 min tap water rinse. The application of Weigert’s hematoxylin lasted 5 min, followed by another 5 min wash with tap water, and a quick rinse in distilled water. The slides were subsequently stained with Biebrich’s scarlet acid fuchsin for 5 min, rinsed in distilled water, and bathed in phosphoric–tungstic–phosphomolybdenum acid for an additional 5 min. A 5 min application of aniline blue was the next step, and finally, the slides were secured in 1% acetic acid for a period of 2 min.

Microscopic examination of the preparations was carried out on a Zeiss AxioLab 4.0 microscope with a magnification of ×400. AxioVision 7.2 software for Windows was used to analyze and photograph the images.

Two independent investigators with expertise in animal models conducted the morphometric study, uninformed of the group each animal belonged to. The terminology used in the histomorphometric analysis adhered to the guidelines provided by the Histomorphometry Nomenclature Committee of the American Society for Bone and Mineral Research [33].

The following parameters were determined:

(1) The percentage of newly formed bone tissue
(2) Histopathological pattern of bone graft remodeling.

The morphometric evaluation of bone tissue was carried out within an area defined radially by the defect’s edges and laterally by the native femur along with the outer boundary of the bone graft and/or the newly generated bone tissue. This estimate was represented as a percentage of the total defect zone area. For each bone defect, three histological sections were analyzed and their average value was computed. Tissues indicative of a non-specific reparative process, such as vessels or bone canal width, were not included in the quantification and constituted the smallest percentage in the bone callus area [34,35].

The process of distinguishing between bone graft remodeling, new bone formation, and differentiation from residual and lysed bone grafts was accomplished through different staining characteristics of bones at various stages of maturity and differences in staining between newly formed bone fragments and the graft. The chromatic distinction between the bone graft, which stained more toward red, and the newly formed bone, which stained blue, was examined using Masson’s trichrome.

A morphometric assessment of graft remodeling was conducted by identifying the dominant histomorphometric pattern of the bone graft within the newly formed bone defect.

Histomorphometric patterns of bone graft remodeling included:

− Positive bone graft remodeling: this involved remodeling with bone integration, graft remineralization, and the circular formation of new bone tissue.
− Negative bone graft remodeling: this represented remodeling with resorption and fibrotic replacement of the bone graft.

**Figure 3:**
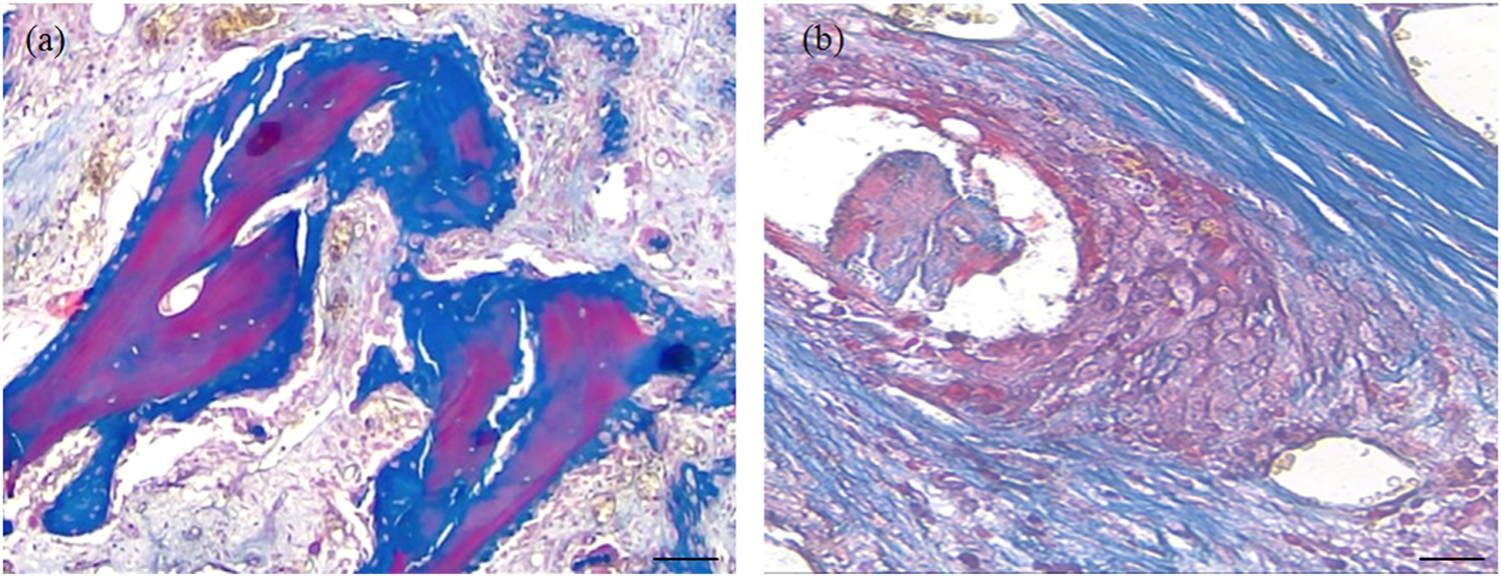
Histopathologic patterns of bone graft remodeling: (a) positive remodeling with integration, remineralization, and circular formation of newly formed bone tissue (Masson’s trichrome × 40. Scale bar, 500 µm) and (b) negative remodeling with resorption and fibrotic replacement of the bone graft (Masson’s trichrome × 40. scale bar, 500 µm).

These patterns of bone graft remodeling are illustrated in Figure 3.

Bone graft remodeling was assessed according to a scale: minimal – less than 10%, moderate – 11–50%, and pronounced – more than 50%.

### 2.5 X-ray imaging

Radiological examinations were conducted 2 h prior to the termination of the animals from the study at the intervals of 14, 30, and 60 days, using frontal and lateral projections on a radiological machine (Platinum, Apelem, France).

The radiological data were analyzed according to the adapted Lane and Sandhu radiograph evaluation method (Table 1) [36]. Average scores were determined for each metric, followed by the aggregation of scores across various metrics (such as periosteal reaction, defect size, presence of new growth in the defect, bone remodeling, and bone allograft remodeling). This cumulative score was used to quantify the degree of healing in each group. The bone formation in each defect was independently scored by three radiologists. The group that achieved the highest overall radiological score was deemed to have experienced the most successful healing.

**Table 1:** Radiological assessment of bone defect healing

No.	Characteristics	Points
1	**Periosteal reaction**	
	Yes	1
	No	0
2	**Remodeling and resorption of the bone graft**	
	No change	0
	Partial	1
	Full	2
3	**Defect size**	
	No change or increase	0
	Decrease of less than 25%	1
	By 25–50% decrease	2
	By 50–75% decrease	3
	More than 75%	4
	No defect	5
4	**Filling bone defect with regenerate**	
	Yes	1
	No	0
5	**Bone remodeling**	
	Complete	2
	Bone marrow canal	1
	No	0
Maximum radiological score		11

### 2.6 Statistical analysis

All experimental values are displayed as mean and standard deviations. Comparisons between two groups were made using the chi-squared test with Yates continuity correction and Mann–Whitney test, while multiple comparisons were made using Pearson’s chi-squared test. Statistical analysis of the research results was performed using IBM SPSS Statistics 20.0 and STATISTICA 10. A *p*-value of <0.05 was deemed to indicate statistical significance.

## 3 Results

### 3.1 Histological and morphometric analyses of newly formed bone tissue

**Figure 4:**
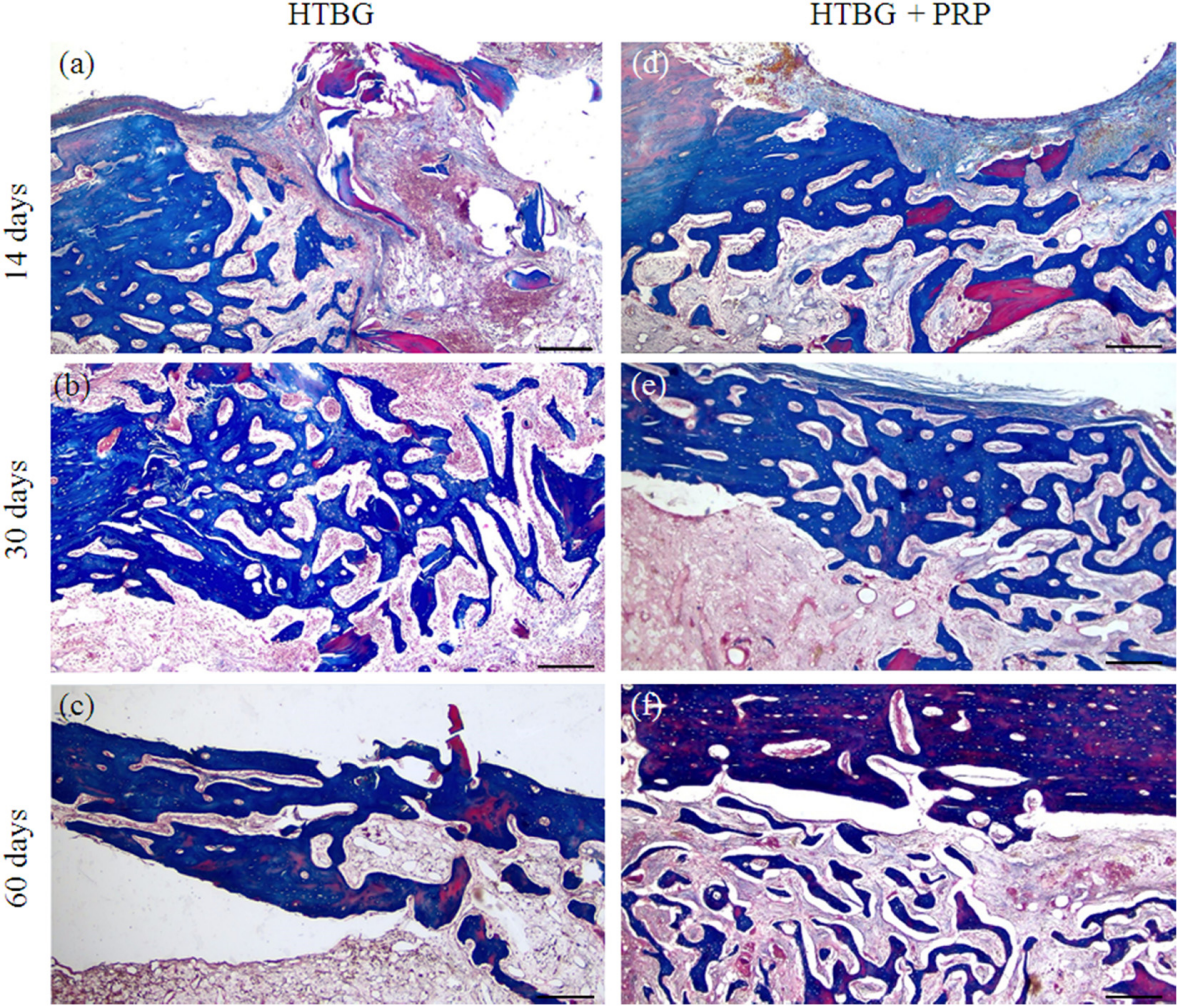
Microphotographs of stage-specific closure of the cortical plate defect in the study groups 14, 30, and 60 days after implantation (Masson’s trichrome × 40. Scale bar, 500 µm) (a–c) – HTBG group: (a) negative remodeling of the bone graft with lysis and resorption; single heterogeneous bundles of newly formed bone tissue are not integrated with the bone graft; (b) lysed bone graft is replaced by fibrous tissue, and the remaining fragments are integrated with newly formed bone beams; and (c) defect area is closed by newly formed bone tissue with foci of disorderly distribution of mature and immature bone matrix; (d–f) – HTBG with PRP (HTBG + PRP group): (d) positive remodeling of the bone graft with bone integration, remineralization, and circular formation of newly formed bone tissue, partial closure of the defect with newly formed bone beams; (e) the osteotomy site is closed with mature bone tissue with multiple wide bridge-like contacts between newly formed bone beams; and (f) the defect area is closed with mature bone tissue.

**Figure 5:**
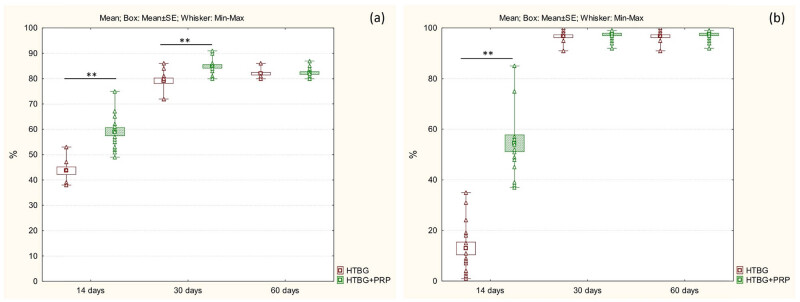
Histological image of newly formed bone tissue and positive bone graft remodeling in the defect area after 14, 30, and 60 days: (a) comparison of the amount of newly formed bone tissue between groups (*p < 0.01, **p < 0.001) and (b) comparison of positive bone graft remodeling between groups (*p < 0.01, **p < 0.001).

On day 14 for the HTBG group, the newly formed bone tissue constituted 41.5% of the total (Figure 5a). The area of the bone defect was characterized by the development of individual heterogeneous bundles of new bone tissue. These bundles contained lacunae with osteocytes and an abundance of vascular channels. The emerging bone beams were uneven, primarily thin, with focal bridging regions and sporadic contacts, mostly at the ends of bone passages (Figure 4a). In the HTBG + PRP group, the new bone formation made up 58% of the total (Figure 5a). There was a progressive increase in the formed bone tissue, featuring haphazardly arranged Haversian canals and widespread, broad bone trabeculae with numerous extensive bridge-like contacts (Figure 4d).

On the 30th day, the newly formed bone tissue in the HTBG group accounted for 80.5% of the total (Figure 5a). The bone tissue consisted of haphazardly arranged bone trabeculae and tendons that were merging into lamellar structures. The bone tract exhibited significant mineralization and robust longitudinal growth (Figure 4b). Meanwhile, in the HTBG + PRP group on day 30, the bone tissue constituted 84.5% of the total (Figure 5a), with the defect area made up of dense mineralized bone tissue containing Haversian canals of varying sizes.

By day 60 in both HTBG and HTBG + PRP groups, the defect site revealed formed bone tissue with normal development of bone trabeculae, predominantly composed of spindle-shaped osteocytes (Figures 4c,f and 5a).

Neither the HTBG group nor the HTBG + PRP group displayed evidence of chondroid or bone callus hyperplasia on the histological sections. Instead, thin layers of fibrous tissue and blood vessels were visible, along with varying extents of bone graft resorption and the presence of fibrovascular structures. These observations were similar across both groups, and no additional histological alterations were attributable to the use of PRP.

### 3.2 Comparative analysis of bone graft remodeling

On day 14 in the HTBG group, less than 20% exhibited positive remodeling of the bone graft, while in the HTBG + PRP group, a statistically significant positive remodeling of the bone graft was observed (Figure 5a and b).

On the 30th day in the HTBG group, most of the lysed fragments of the bone graft had been replaced by fibrous tissue, while positive bone graft remodeling was seen in all the remaining fragments. For the HTBG + PRP group, the tissue composition consisted of remaining bone graft fragments closely interacting with the newly formed bone and osteoid tissue. There was noticeable active integration of the bone allograft with the new bone and pronounced positive bone graft remodeling (Figure 5b). No statistically significant difference was observed between the groups at this point.

By day 60 in both HTBG and HTBG + PRP groups, newly formed bone tissue without bone graft fragments was seen at the defect site. However, the histological material in the HTBG group also revealed areas with osteoid tissue (forming bone tissue) and regions with a random distribution of mature and immature bone matrix, while the HTBG + PRP group exhibited a consistent and uniform distribution of mature bone tissue (Figure 5b).

### 3.3 Comparative characterization of inflammatory and necrotic pattern

At 14 days in both HTBG and HTBG + PRP groups, a single reactive cell infiltrate without necrotic changes was observed at the defect site. At 30 and 60 days, all regions of surgically created defects showed no signs of inflammatory cell infiltration, infection indicators (polymorphonuclear cells, lymphocytes, macrophages, and multinucleated cells), or tissue necrosis.

### 3.4 Radiographic evaluation

**Figure 6:**
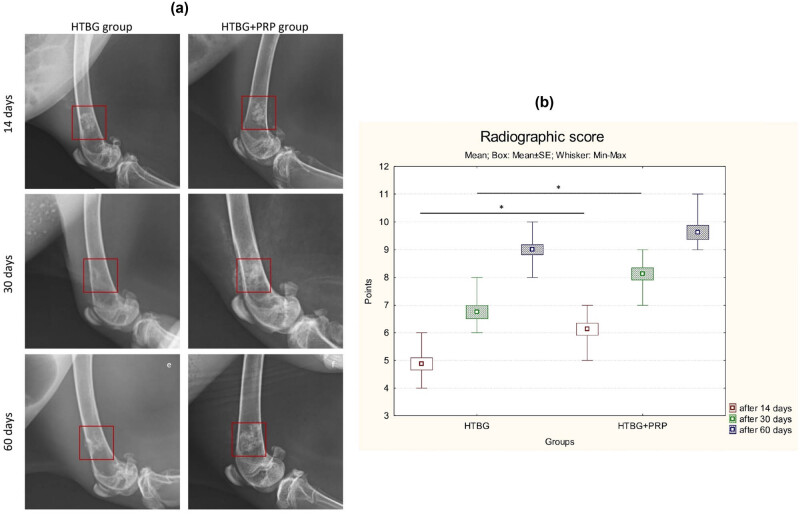
Radiographic assessment of femoral bone defect healing: (a) radiographic images after 14, 30, and 60 days and (b) comparison radiographic score between groups (*p < 0.05).

The lateromedial X-ray images illustrated distinct areas of radiolucent bone defect in the femur of all animals after a 2 week period (Figure 6a). The boundaries of the scaffolds began to blur, and a high-density signal appeared on the periphery of the bone defect, suggesting new bone formation in the affected areas for both groups. Notably, the HTBG + PRP group demonstrated a statistically significant (*p* < 0.01) enhancement in the radiographic score in comparison with the HTBG group after 14 days (Figure 6b).

By the end of 30 days, the materials implanted in the bone had been substituted by newly grown bone, partially filling in the bone defects (Figure 6a). The defect areas in the bone were largely unobservable in both groups. There was an increased radiopacity in the femur where HTBG + PRP was implanted, demonstrating a higher radiographic score than the adjacent bone and notably greater than that in the HTBG group (Figure 6b).

At the 60 day mark, the areas of bone defect were no longer discernible in either group. All groups exhibited enhanced radiopacity in the femur, with a non-significant uptick in radiographic score (Figure 6b).

## 4 Discussion

This study provides an examination of bone graft remodeling and the healing pattern of newly formed bone following the application of the Marburg bone graft with and without PRP, based on an animal model experiment. The evaluation incorporates radiological, histopathological, and histomorphometric assessments.

Our findings demonstrate that the use of PRP with thermally disinfected bone graft significantly enhances bone formation when compared to the group using bone graft alone, without additional biocomponents. Particularly, the PRP group displayed a notably larger volume of newly formed bone tissue and higher radiologic scores on the 14th and 30th day, compared to the group without PRP (*p* < 0.001). Furthermore, the defect area in the PRP group revealed a more pronounced proliferation of mature bone tissue that was integrated with the allograft, and it showed the presence of chaotically arranged Haversian canals and horizontally expanding bone trabeculae. The results we obtained supplement the existing evidence demonstrating that the combination of PRP and thermally treated bone is just as effective as the combination of PRP with other graft materials. Several researchers [22,33] have suggested that combining PRP does not necessarily result in a significant boost in osteogenesis. This could potentially be due to high-temperature treatment of the biomaterial causing irreversible changes, which may diminish the impact of growth factors present in PRP [37]. Drawing on our findings, we postulate that using PRP with heat-treated bone enhances the initial stage of osteogenesis. Consequently, the early phase of fracture healing with a Marburg bone graft could be expedited through appropriate growth factor stimulation, via the synergistic effect of PRP. This process might lower the occurrence of non-unions and infections.

Previously, it was shown that PRP improves the regenerative properties of connective and epithelial tissue by increasing the activity of fibroblast-like cells and stimulating cell proliferation [38–42]. We believe that bone graft in combination with PRP improves osteoconductive potential and induces an osteoinductive effect, which is reflected in the enhancement and acceleration of growth and maturation of bone tissue in the defect area.

On the 14th and 30th day, the group using PRP showed a dominant pattern of positive bone graft remodeling in the histopathological analysis, revealing improved viability of the bone graft and a greater proportion of mature bone matrix compared to the group using only bone graft. Despite being a commonly used and convenient method, bone repair with bone grafts has some drawbacks, such as poor bone graft viability and negative remodeling of the bone graft. These issues can lead to relative delays in bone repair or disjointed, uneven healing of the bone defect. This gradual replacement process might result in persistent zones of bone necrosis [43]. Our findings suggest that PRP can effectively diminish the resorption of a thermally treated bone graft, bolster the integration of newly formed bone with the graft surface, and expedite the bone graft remodeling process. This has promising implications not only for bone grafting, but also for other orthopedic scenarios requiring bone remodeling with minimized necrosis risks.

In earlier studies, PRP has been shown to improve angiogenesis in the area of bone defects [44–46]. The process of angiogenesis is significant and necessary for tropism, growth, and maturation of bone tissue [45,47]. Previously [44], we found that in groups, the formation of more dense zones of maturing and mature bone tissue was observed in zones with a relatively large number of microvessels. We believe that the PRP-induced effect of bone growth and maturation activity is more likely associated with a direct effect on angiogenesis and indirectly on an osteoinductive effect.

Our investigation revealed that, as per the histological and radiological findings, there was no significant difference in bone healing between the HTBG and HTBG + PRP groups at the end of 60 days. We propose that this lack of difference may be attributed to the completion of the active phase of osteogenesis, resulting in complete closure of the bone defect. However, it is important to acknowledge that bone tissue reconstruction and remodeling are complex processes that continue beyond the active phase of osteogenesis [1,6]. These processes involve the reorganization and reshaping of the newly formed bone tissue, ensuring its strength and functionality. Although complete closure of the bone defect may have been achieved, the ongoing reconstruction and remodeling could still be taking place [17,42]. This ongoing process might contribute to the lack of significant difference observed at day 60. This suggests the need for additional research to fully understand these processes.

As previously demonstrated, approximately 90% of the bone lamina is composed of mature lamellar bone tissue, with the remaining 10% comprising lacunae, Haversian canals filled with vessels and nerve fibers, as well as fibrous and cartilaginous tissues [48]. The lamellar structure of the bone lamina, integrated with a system of channels, offers not just nutritional support but also mechanical resilience, as bone’s resistance to deformation relies not merely on its mineral content but also on its intricate internal microstructure, a crucial outcome of successful defect repair. Bone tissue repair conventionally transpires in a stage-specific manner, encompassing a phase of dynamic growth and a maturation phase distinguished by the emergence of channels for nutrition and trophicity. This maturation phase is signaled by a relatively minor decline in bone tissue quantity due to focal resorption. According to our study, the bone plate in the defect zone for the HTBG + PRP group at 60 days displayed a minor reduction in bone tissue quantity. This aligns with the process of bone reconstruction and the formation of Haversian canals and may be perceived as a stage of bone tissue maturation [49]. Conversely, in the group without PRP, at 60 days, there was a tendency toward an increase in bone tissue, accompanied by regions displaying haphazard distribution of immature bone matrix. This suggests an extended potential for bone growth and a relative delay in maturation. We posit that the use of PRP with a HTBG not only enhances the osteoconductive and osteoinductive potential of the graft, but also expedites the maturation process of the newly formed bone tissue.

Furthermore, we showed that in the PRP group, there were no abnormalities in bone repair associated with insufficient or excessive bone formation. Newly formed bone tissue in both groups was characterized by normal histopattern: (1) the bone plate did not extend beyond the thickness of the bone defect and did not pass to the bone plate outside the defect, (2) had a laminar layered structure with a focal chaotic pattern in the area of predominantly Haversian canals, and (3) the relative amount of bone tissue and Haversian canals did not differ both between groups and from the structure of the bone outside the bone defect.

A key advantage of this study lies in the comparative evaluation of using a Marburg bone graft in conjunction with PRP, during both early and late phases of bone defect healing. This approach facilitated the discovery of osteoregeneration stimulation in the early stages through the use of PRP. The use of a standardized rabbit femoral defect model, alongside histological and morphometric analysis, further strengthens the validity of the study.

Limitations: The precise mechanism of PRP’s impact on bone tissue is still subject to debate. Is its role direct, influencing osteosynthesis and bone maturation, or does it indirectly promote a favorable macroenvironment with a high index of angiogenesis? These queries could be addressed in subsequent research. Additionally, the evaluation of bone regeneration relied solely on histological and radiographic analyses, which, although informative, did not fully exhaust all the potential analytical methods. Techniques like micro-computed tomography and immunohistochemistry were not used. These could have contributed to a more well-rounded comprehension of bone regeneration by scrutinizing the structural aspects, cellular elements, and gene expression profiles of the regenerated bone. These limitations should be considered when interpreting the results and should influence the design of future studies to fill these potential knowledge gaps.

## 5 Conclusions

Thus, our experiment with rabbits demonstrated that the combined application of Marburg bone grafts and PRP enhances the viability of the bone graft within the defect zone. We found that PRP improves the integration of the graft with the bone and accelerates the remodeling process of the bone graft. We believe that the primary effect of enhancing the osteoconductive and osteoinductive potentials, as well as the improvement of bone graft remodeling, is tied to the formation of a locally favorable microenvironment with active perfusion–diffusion potential within the stromal framework. This in turn encourages more active growth and maturation of the bone tissue.
